# Supplementary material for: Viral nucleoprotein antibodies activate TRIM21 and induce T cell immunity
Source: EMBO J. 2020 Dec 1;40(5):e106228. doi: 10.15252/embj.2020106228 (PMC7917548; doi:10.15252/embj.2020106228)
Supplement: Supplementary file 1 — Expanded View Figures PDF [file EMBJ-40-e106228-s001.pdf]

Expanded View Figures

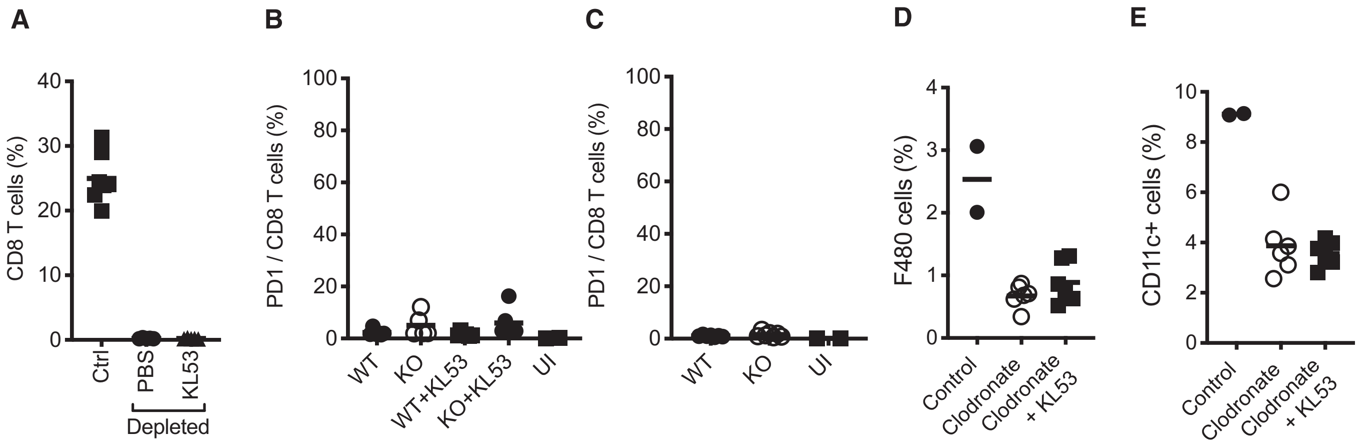

Figure EV1. Extended CTL analysis from WT and KO mice infected with LCMV.

A Effective depletion of CD8 T cells in the spleen 9 days post anti-CD8 mAb administration, in mice infected with LCMV and receiving either KL53 or PBS days 1 and day 3pi.

B, C Surface expression of PD-1 on CD8 T cells of WT and KO mice either (B) day 8pi in the presence or absence of KL53 or (C) day 10pi in naïve mice only.

D, E Effective depletion of macrophages in the spleen using clodronate liposomes, as measured by surface expression of F480 (D) and CD11c (E) by flow cytometry.

Data information: Each data point represents once mouse from the same experiment. The horizontal bars correspond to the mean ± standard error.

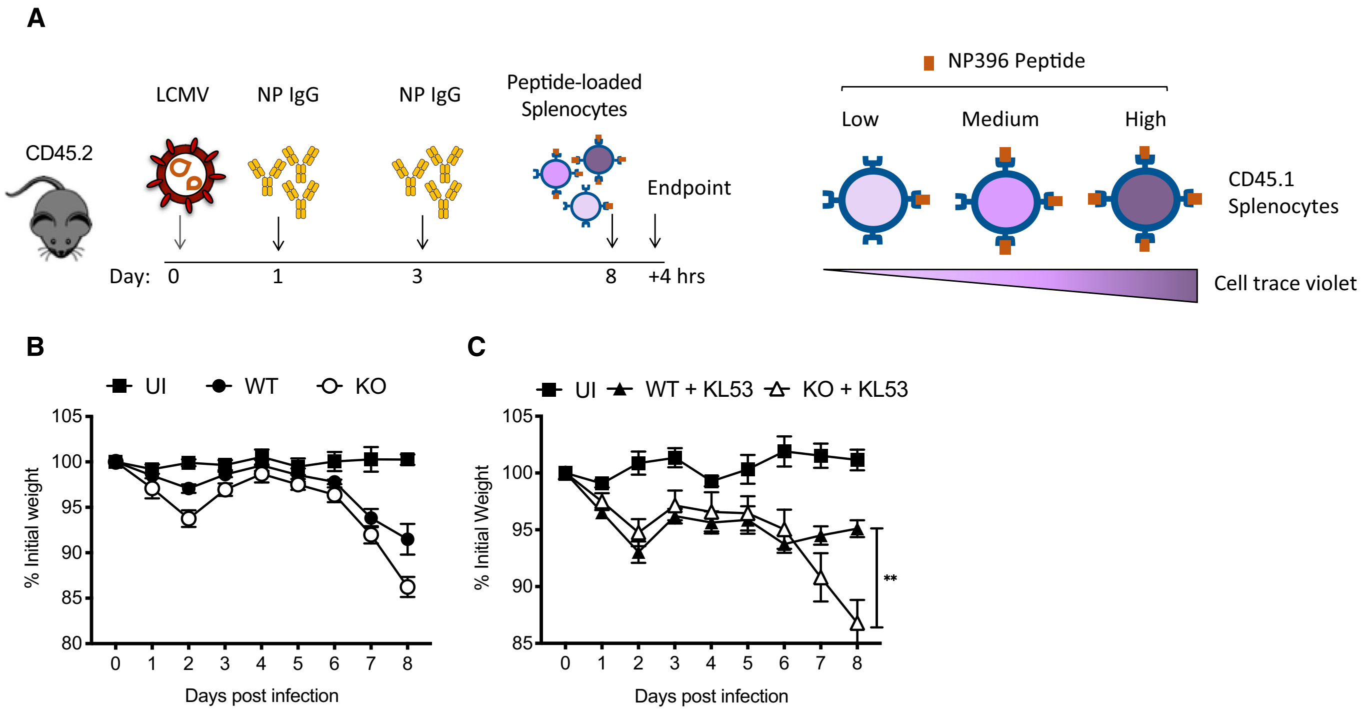

Figure EV2.

◀ **Figure EV2. Experimental detail of *in vivo* killing assay.**

A Timeline of *in vivo* killing protocol, and diagram of pulse-labelling of CD45.1 splenocytes with different concentrations of N_{396} peptide and staining with cell trace violet.
B Weights of WT and KO mice infected with LCMV which received pulsed-labelled splenocytes 8dpi for *in vivo* killing experiment shown in Fig 4A.
C Weights of WT and KO mice infected with LCMV then passively transferred with KL53 that received pulsed-labelled splenocytes 8dpi for *in vivo* killing experiment shown in Fig 4B.

Data information: All data are presented as mean with standard error ($n = 6$). $^{**}P < 0.01$, unpaired t-test.

 © 2020 The Authors
