# Supplementary figures and images for: Viral nucleoprotein antibodies activate TRIM21 and induce T cell immunity
Source: EMBO J. 2020 Dec 1;40(5):e106228. doi: 10.15252/embj.2020106228 (PMC7917548; doi:10.15252/embj.2020106228)

WB for LCMV N protein

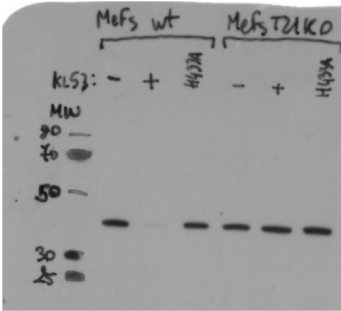

WB for Vinculin

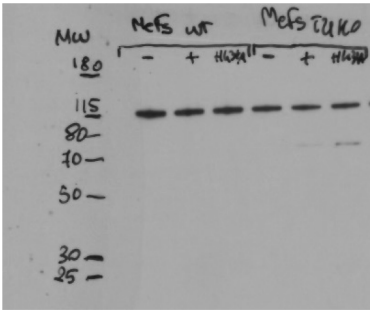

Supplement: Supplementary file 3 — Source Data for Figure 1 [file EMBJ-40-e106228-s002.pdf]
